# Cybernic robot hand-arm that realizes cooperative work as a new hand-arm for people with a single upper-limb dysfunction

**DOI:** 10.3389/frobt.2024.1455582

**Published:** 2024-10-22

**Authors:** Hiroaki Toyama, Hiroaki Kawamoto, Yoshiyuki Sankai

**Affiliations:** ^1^ Center for Cybernics Research, University of Tsukuba, Tsukuba, Japan; ^2^ CYBERDYNE, Inc., Tsukuba, Japan; ^3^ Faculty of Engineering, Information and Systems, University of Tsukuba, Tsukuba, Japan

**Keywords:** assistive robots, single upper-limb dysfunction, cooperative work, human–machine interaction, cybernics, cybernic robot hand-arm

## Abstract

A robot hand-arm that can perform various tasks with the unaffected arm could ease the daily lives of patients with a single upper-limb dysfunction. A smooth interaction between robot and patient is desirable since their other arm functions normally. If the robot can move in response to the user’s intentions and cooperate with the unaffected arm, even without detailed operation, it can effectively assist with daily tasks. This study aims to propose and develop a cybernic robot hand-arm with the following features: 1) input of user intention via bioelectrical signals from the paralyzed arm, the unaffected arm’s motion, and voice; 2) autonomous control of support movements; 3) a control system that integrates voluntary and autonomous control by combining 1) and 2) to thus allow smooth work support in cooperation with the unaffected arm, reflecting intention as a part of the body; and 4) a learning function to provide work support across various tasks in daily use. We confirmed the feasibility and usefulness of the proposed system through a pilot study involving three patients. The system learned to support new tasks by working with the user through an operating function that does not require the involvement of the unaffected arm. The system divides the support actions into movement phases and learns the phase-shift conditions from the sensor information about the user’s intention. After learning, the system autonomously performs learned support actions through voluntary phase shifts based on input about the user’s intention via bioelectrical signals, the unaffected arm’s motion, and by voice, enabling smooth collaborative movement with the unaffected arm. Experiments with patients demonstrated that the system could learn and provide smooth work support in cooperation with the unaffected arm to successfully complete tasks they find difficult. Additionally, the questionnaire subjectively confirmed that cooperative work according to the user’s intention was achieved and that work time was within a feasible range for daily life. Furthermore, it was observed that participants who used bioelectrical signals from their paralyzed arm perceived the system as part of their body. We thus confirmed the feasibility and usefulness of various cooperative task supports using the proposed method.

## 1 Introduction

Some people suffer paralysis in one of their upper limbs because of damage to the brain or nervous system or other causes and are forced to live with one arm (Wade et al., 1983; Rosson, 1987; Backe et al., 2008). Those with a single upper-limb dysfunction daily encounter tasks that are difficult or impossible for them owing to the limitation of having only one functional arm (Sköld et al., 2004; Mancuso et al., 2015). Moreover, the load concentration on the unaffected arm causes fatigue and decreases the daily work they can perform. These represent significant barriers to everyday life and social participation.

One currently available measure is the use of welfare equipment (Performance Health, 2024; Etac, 2024). However, none of these tools can provide active support, such as the ability to move like a human arm. Consequently, all tasks must be performed with only one unaffected arm, so load concentration cannot be eliminated. In addition, it is difficult to carry and use all assistive devices for each task in diverse work environments. Moreover, tasks requiring the simultaneous movement of both arms, such as tipping a jelly cup while eating its contents, are difficult to execute. To solve the source of this problem, an alternative to the paralyzed arm would be necessary. A robot hand-arm that can grip daily necessities and perform various tasks in cooperation with the patient’s unaffected arm offers a solution to the limitations described above.

**FIGURE 1:**
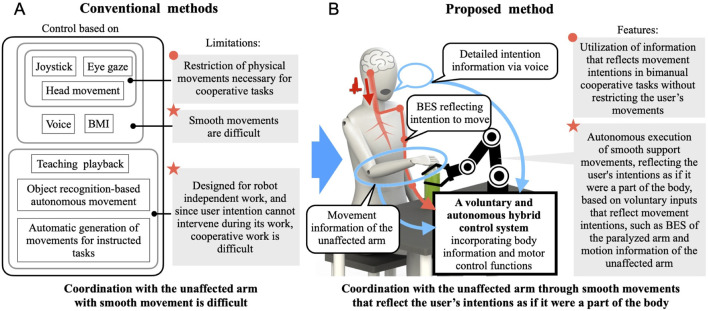
Issues of conventional methods for cooperative work with the unaffected arm and solution by the proposed method. **(A)** Conventional methods. **(B)** Proposed method.

Research and development have been conducted on a robot hand-arm attached to a wheelchair for people with upper-limb dysfunction (Hillman et al., 2002; Romer et al., 2005; Alqasemi and Dubey, 2007; Maheu et al., 2011; Wakita et al., 2012). However, these robot hand-arms for disabled users perform movements by operating a controller such as a joystick. Therefore, when used by a patient with a single upper-limb dysfunction, the unaffected arm is restrained for the operation, and it is also difficult for these robot hand-arms to perform cooperative work with the patient’s unaffected arm. Alternative methods of operation, such as chin-operated joysticks and controllers using head movements, exist for individuals with bilateral upper-limb dysfunctions (Fall et al., 2015; Aspelund et al., 2020; Rulik et al., 2022). However, it is inconvenient for patients with a single upper-limb dysfunction to have their head movements restricted to these operations, as bimanual tasks often require head movements such as turning the face to the left or right work area based on the arm moving to pick up an object, or adjusting face direction to ensure that the hands performing the task are in the center of the field of vision. There is also an eye-gaze-based method, but similar problems occur (Scalera et al., 2021; Sunny et al., 2021). It is desirable that the physical functions necessary for the task, including the unaffected arm, are not restricted when a robot hand-arm cooperates with a patient’s unaffected arm. While brain–machine interfaces using brain activity signals also exist, fine operation and smooth movements are difficult due to limitations in resolution and the complexity of the information (Kim et al., 2015; Karunasena et al., 2021; Peng et al., 2022). Furthermore, these systems tend to be bulky, making them impractical for patients with a single upper-limb dysfunction to carry and use in daily life. Invasive methods also carry surgical risks, making them less accessible. There is a voice-based method (Poirier et al., 2019), but all the methods described so far, including voice-based, require complex operations to control the robot hand and its position, orientation, and so on. When both upper limbs are affected, it is effective to operate a robot hand-arm to perform tasks, even if it takes a long time. However, for patients with a single upper-limb dysfunction, smooth interaction between the robot hand-arm and the patient is desirable, as their other arm functions normally. Methods to operate a robot hand-arm without requiring complex operations currently include teaching playback, object recognition-based pick-and-place, and automatically generating movements for instructed tasks (Ahn et al., 2022; Wohlgemuth et al., 2024; Shahria et al., 2024). However, these methods are designed for the robot hand-arm’s independent operation. In these approaches, it is difficult to reflect human intent in the robot’s movements during the task, making it difficult to coordinate with the unaffected arm during tasks. As described above, conventional robot hand-arms for persons with upper-limb dysfunctions are designed for individuals with bilateral upper-limb dysfunction, and tasks are performed only by the robot hand-arm. No method has been designed to support patients with a single upper-limb dysfunction, and there is no method that enables smooth coordination with the unaffected arm. Figure 1A summarizes the issues faced by conventional methods for cooperative work with the unaffected arm. This study aims to address these issues and achieve smooth cooperative work between the robot hand-arm and the unaffected arm according to the patient’s intention.

For the robot hand-arm to be able to replace the paralyzed arm and perform cooperative tasks with the unaffected arm, it should move according to the user’s intention, similar to a body part, and simultaneously perform the corresponding actions alongside the unaffected arm. For a robot hand-arm to perform such actions, it is necessary to input information that reflects the user’s intention for movement in cooperative work involving both hands. If the robot hand-arm can automatically perform support actions based on this input information, smooth cooperative work can be achieved. On the other hand, this information input should not disturb the user’s activities, such as unaffected arm and head movements. In this study, the robot hand-arm served as a replacement for the paralyzed arm. Therefore, as long as the information reflects the intention to move obtained from the paralyzed arm, it is possible to input intuitive intention information according to the motion that the user desires the robot hand-arm to perform without disturbing the user’s other physical movements. Although the patient’s paralyzed arm is unable to perform tasks because its motor functions are limited owing to the paralysis, it is possible to capture the paralyzed arm’s intention to move from the bioelectrical signal (BES) that communicates the intention to move from the brain (Sankai, 2011; Saita et al., 2018). Additionally, since cooperative work involves actions performed by one arm in conjunction with the other, information on corresponding actions can be obtained from the motion of the unaffected arm during tasks. Furthermore, using voice information, the tasks and detailed movements that the user wants to perform can be captured without limiting the patient’s movements, covering cases where movement intentions or detailed movement information are difficult to capture with the BES of the paralyzed arm or the movement information of the unaffected arm. By using BES, motion information from the unaffected arm, and voice information, it is possible to realize robot hand-arm movements according to the user’s intentions. Therefore, we propose a cybernic robot hand-arm that is connected to the user via this information and functions according to the user’s intention information with a combination of voluntary and autonomous control. This allows the autonomous execution of movements such as fine adjustment of gripping force and hand movement corresponding to the task (Figure 1B). By incorporating the user’s body information and motor control functions into the control system, the cybernic robot hand-arm can become part of the user’s body and smoothly perform tasks alongside the unaffected arm, reflecting the user’s intentions. Even intention information that is not applicable to detailed operations can be used as input in such a control system. Moreover, if a cybernic robot hand-arm can learn the intention information input to the system and the support actions according to the task during application in daily life, it will be possible to smoothly support various cooperative tasks that are repeatedly performed in daily life.

The purpose of this study is to propose and develop a cybernic robot hand-arm with the following features to address the difficulties faced by patients with a single upper limb dysfunction in performing daily tasks: 1) input of BES that reflects the intention to move from the paralyzed arm, motion information from the unaffected arm, and voice-based intention information; 2) autonomous control of support movements according to the unaffected arm and work phase; 3) a control system that integrates voluntary and autonomous control by combining 1) and 2) allowing for smooth work support in cooperation with the unaffected arm, reflecting intention as a part of the body; and 4) a learning function to provide such work support across various tasks in daily use. We confirmed the feasibility and usefulness of the proposed method through a pilot study involving three patients.

## 2 Cybernic robot hand-arm

### 2.1 Overview of the proposed method

The proposed system is not a substitute for the patient’s difficult bimanual tasks but instead supports these tasks in cooperation with the unaffected arm, complementing the role of the paralyzed arm. We define “work support” as the support provided for bimanual tasks in cooperation with the unaffected arm. The features of the proposed method are as follows: 1) input of BES that reflects the intention to move from the paralyzed arm, motion information from the unaffected arm, and voice-based intention information; 2) autonomous control of support movements according to the unaffected arm and work phase; 3) a control system that integrates voluntary and autonomous control by combining 1) and 2), allowing for smooth work support in cooperation with the unaffected arm, reflecting intention as a part of the body; and 4) a learning function to provide such work support across various tasks in daily use.

**FIGURE 2:**
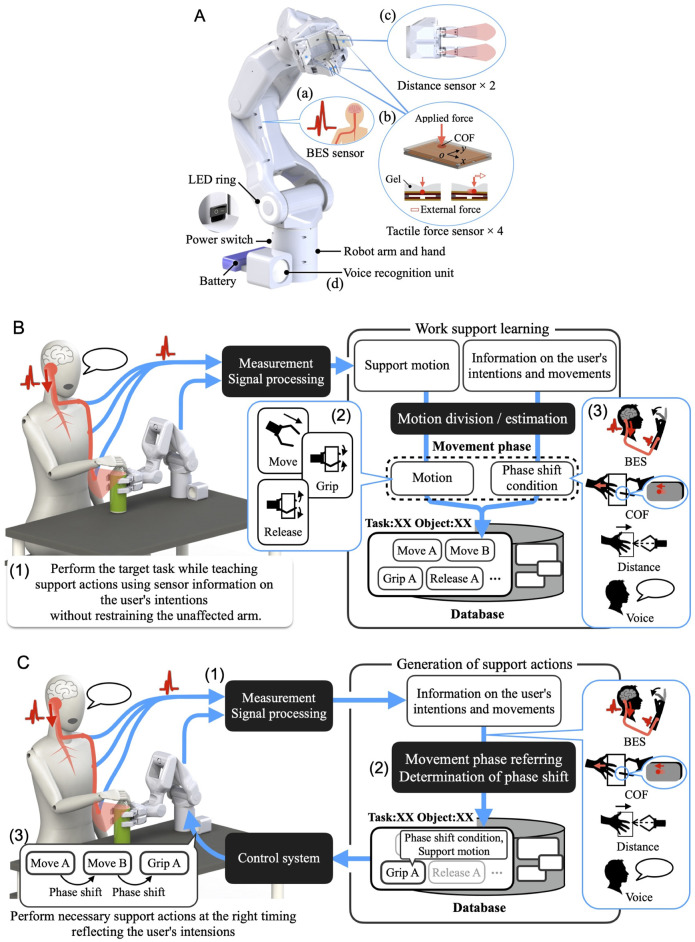
**(A)** Overview of the cybernic robot hand-arm. **(B)** Overview of the work support learning that reflects the user’s intention and cooperates with the unaffected arm. **(C)** Overview of the work support based on learned data that cooperates with the unaffected arm according to the user’s intentions.

We first developed a robot hand-arm system capable of acquiring and linking the information on the user’s intentions and movements (Figure 2A). A sensor system was installed to measure the BES of the paralyzed arm as intuitive intention information reflecting the paralyzed arm’s intention to move (Figure 2A(a)). We incorporated a sensor system in the robot hand to capture the movement information for the unaffected arm, considering the characteristics of cooperative work where both hands interact with an object. These include tactile force sensors to detect the direction of force applied by the unaffected arm on the object gripped by the system and distance sensors to capture the motion of the unaffected arm moving the work object closer to or away from the system’s hand (Figure 2A(b) (c)). Additionally, to cover detailed movement and task information that may be difficult to capture through the BES of the paralyzed arm or movement information from the unaffected arm, we also developed a voice recognition unit (Figure 2A(d)). We developed a portable robot hand-arm system equipped with these sensor systems that is about the same size as a human and can be used for cooperative work with the unaffected arm in daily life.

Cooperative work involving both hands comprises movement phases and phase shifts, such as moving, gripping, and releasing one hand in correspondence and coordination with the other hand. Therefore, we developed a work support learning and control function. The system learns to support new tasks by working with the user through an operating function that does not require the unaffected arm (Figure 2B(1)). The system divides the support actions into movement phases and learns the phase-shift conditions from the sensor information about the user’s intention and motion (Figure 2B(2) (3)). During the learned task support, when the input of the user’s intention information via BES from the paralyzed arm, unaffected arm’s motion, and voice is determined to satisfy the phase-shift condition (Figure 2C(1) (2)), phase shifts and corresponding actions are performed at any time (Figure 2C(3)). This enables the system to autonomously perform learned support actions through voluntary phase shifts based on input related to the user’s intentions and movements, thereby realizing seamless cooperative work and work support that correspond to and are linked to the user’s intentions and the unaffected arm without requiring detailed operations. The key feature of this method is that by incorporating the user’s physical information and motor control function into the control system—such as BES from the paralyzed arm and motion information from the unaffected arm—the system becomes part of the user’s body and can smoothly perform cooperative work with the unaffected arm through autonomous motions that reflect the user’s intentions.

Details of the robot hand-arm system are provided in Section 2.2, while details of the learning and control function are described in Section 2.3.

### 2.2 Robot hand-arm system

This section describes the reasons for determining the specifications and provides details of the hand-arm and sensor systems shown in Figure 2A.

#### 2.2.1 Robot hand-arm

The specifications of the robot hand-arm were determined assuming the support of a tabletop for the patients. The paralyzed side of the patient is vulnerable to joint dislocation due to muscle weakness and relaxation, as well as sensory disturbances such as numbness and pain (Raghavan, 2015). Consequently, the robot hand-arm was designed to be placed on a tabletop to accommodate these factors. It can be easily attached there with a clamp. To ensure portability and compatibility with the unaffected arm without obstructing surrounding objects or limiting the workspace, the arm’s size is approximately the same as a human arm. This enables the robot to be applied to the movements performed by the paralyzed side as well as to the living environment where the space is already available for work with both hands. Assuming work support for a patient whose unaffected arm became the dominant hand, the arm part of the device has six degrees of freedom to support work in various positions and postures, and the hand part has two sets of two fingers facing each other to enable pinching with two fingers and stable gripping with four fingers, enabling the system to perform tasks that are mainly performed by the non-dominant hand such as gripping, fixing, and moving objects. An analysis of functionality and disability in everyday life using the International Classification of Functioning, Disability, and Health (ICF) revealed that lifting items weighing 0.4 kg or more accounts for approximately 10% of lifting actions in daily life (Shiraishi et al., 2010). Hence, if the system can lift an object weighing 0.4 kg, approximately 90% of daily activities can be performed. In this study, we included a 500-mL PET bottle as a work object, necessitating a payload capacity of 0.6 kg. Specialization for the targeted movements and the monocoque structure have enabled the development of a compact and lightweight system with a reach of 619 mm and a total weight of 2.9 kg, including the sensor system.

#### 2.2.2 Sensor system for capturing intentions and motion information

The proposed method employs a control system that combines voluntary and autonomous control for performing movements in coordination with the unaffected arm, using, as input, information that reflects the intention to move both hands cooperatively. We determined the specific intention and motion information to be acquired and developed and integrated a sensor system for its acquisition, considering the characteristics of patients with a single upper-limb dysfunction and cooperative work with the unaffected limb.

In this study, the robot hand-arm served as a replacement for the paralyzed arm. Therefore, as long as the information we utilized reflected the intention to move obtained from the paralyzed arm, it would be possible to input intuitive intention information that follows the motion that the user wants the robot hand-arm to perform without disturbing the user’s other physical movements. Although the patient’s paralyzed arm was unable to perform tasks because its motor functions were limited owing to paralysis, it would be possible to capture the paralyzed arm’s intention to move from the ability of the BES to reflect the intention to move from the brain (Sankai, 2011; Saita et al., 2018). Even when paralysis makes joint movement difficult, it is possible to detect weak neuromuscular activity from the BES and estimate the intention to move (Saita et al., 2018). The BES that reflects the intention to move from the user’s brain to the target part of the body can be measured by attaching electrodes to that part for which the system wants to estimate the user’s intention to move. Therefore, a BES measurement circuit is installed inside the system to estimate the intention to move the paralyzed arm (Figure 2A(a)).

Additionally, since cooperative work involves actions performed by one arm in conjunction with the other, information on corresponding actions can be obtained from the motion of the unaffected arm during tasks. This information can be acquired by attaching sensors, such as motion sensors, to the unaffected arm. However, it is difficult for the target patients to attach sensors to the unaffected arm by themselves. Although placing a sensor in the working environment to capture the movements of the unaffected arm is another possible approach, in order to reduce the number of items to carry and the effort required for setup, we considered a method that could be completed with just the hand-arm system. Leveraging the characteristic of cooperative work where both hands interact with the target object, we developed a sensor system that acquires the motion information of the unaffected arm through the object via the robot hand. For situations where the system is gripping an object, a tactile force sensor capable of measuring force and the center of force (COF) (Toyama et al., 2020) is embedded in the tip link of each robot finger to detect how the gripped object is pulled by the user’s arm (Figure 2A(b)). The finger surfaces are made of gel sheets with a human skin-like softness, and the surfaces are coated with polyurethane resin with a high coefficient of friction. By capturing movements of the COF on the surface of each of the two opposing fingers caused by the gel-sheet deformation, the system can detect the direction of the force applied by the unaffected arm to the gripped object, such as rotation or pulling movements. This allows it to capture the movement information of the unaffected arm that cannot be visually detected. We confirmed that this method enables the acquisition of specific movements of the unaffected arm that are useful for the cooperative work between the robot’s hand-arm and the user’s (Toyama et al., 2019). This finger with the sensor also provides flexible contact with the object and adjusts the gripping force. For situations where the unaffected arm is gripping an object, distance sensors are mounted between the two fingers facing each other to capture the motion of the unaffected arm moving the object closer to or away from the system’s hand (Figure 2A(c)). Dynamic changes in distance values capture the motion of the unaffected arm. These sensors are also used to determine the gripping form according to the object. The system determines the gripping form as follows: if both distance values fall within the system’s gripping range, four fingers are used for gripping; if only one distance value is within the range, two corresponding fingers are used.

Some intentions and fine movement information may be difficult to capture from the BES of the paralyzed arm or the motion information of the unaffected arm. In such cases, voice input can cover such information without limiting the patient’s movements; it can also recognize detailed information such as what kind of task support or supportive actions the user is requesting, as well as the name of the object to be handled. Therefore, we developed and integrated a voice recognition unit (Figure 2A(d)) which includes a microphone, speaker, and language processing system capable of recognizing input speech information and reading out generated character strings. The unit also provides feedback to the user, such as requesting work support instructions for unlearned tasks. Google assistant SDK was utilized for speech recognition and text-to-speech.

### 2.3 Learning and control function for work support that reflects intention and cooperates with the unaffected arm

Patients face difficulties in performing daily tasks, with variations among them in the types of tasks, target objects, and required support actions. Sensor information obtained during work support, such as movement data from the unaffected arm and BES readings from the paralyzed arm, is assumed to differ based on the user’s motion and degree of paralysis. To address these, we developed a function that enables the system to learn support actions that constitute work support and their implementation conditions from sensor information about the user’s intentions and movements; it does this by actually performing cooperative work while the user is teaching support actions with an operation function which does not rely on the unaffected arm.

The overview of work support learning is depicted in Figure 2B. Learning is conducted for each individual task, such as opening a PET bottle. The task name is recognized through the user’s voice input, and if the task has not yet been learned, the learning process begins. The learning is conducted as the user performs the task with the system using an operating function that does not rely on the unaffected arm (Figure 2B(1)). This operating function is achieved using voice and BES from the paralyzed arm, with further details provided in the following subsection. The taught series of support actions is divided into three movement phases based on the type of movement: move, grip, and release (Figure 2B(2)). For the phase-shift conditions for each movement phase that constitute the learned work support, the following sensor information on the user’s intention and motion can be utilized within this learning framework: BES from the unaffected arm, COF on the surface of the robot finger and distance information between the robot hand and the work object as motion information of the unaffected arm, and voice information (Figure 2B(3)). The change in the sensor information before and after each movement phase serves as the phase-shift condition. The details are provided in the following subsection. Consequently, the user’s voice and the BES of the paralyzed arm are used in two situations: operation for motion teaching and conditions for performing learned support actions.

The overview of work support after learning is depicted in Figure 2C. The selection of learned data is based on the task name recognized from voice information. During work support, the BES of the paralyzed arm, motion information of the unaffected arm, and voice information are collected as information on the user’s intention and motion, as in the case of learning (Figure 2C(1)). Once this information meets the phase-shift condition (Figure 2C(2)), the phase shifts and corresponding actions (support actions) are executed at any time (Figure 2C(3)). This allows for learned support actions to be executed through a voluntary phase shift based on the user’s intentions and movements, thereby realizing seamless cooperative work and work support that correspond to and are linked to the user’s intentions and the unaffected arm without necessitating detailed operations. By incorporating the user’s physical information and motor control function into the control system, such as the BES from the paralyzed arm and the motion information of the unaffected arm, the system becomes a part of the user’s body and can smoothly perform cooperative work with the unaffected arm to reflect intention as a part of the body. Moreover, by learning the intention information input to the system and the support actions according to the task during application in daily life, it becomes possible to smoothly support various cooperative tasks that are repeatedly performed in daily life.

In the following subsections, we describe in detail the operating function that does not rely on the unaffected arm, the division of support actions into movement phases, and the phase-shift condition learning in this function.

#### 2.3.1 Operating functions using information about intention

When teaching support actions to the system, the unaffected arm of the patient cannot be utilized, as it works in conjunction with the system. Additionally, the user must be capable of operating the system in detail, including controlling the movement position and posture of the system’s hand. Furthermore, intuitive operation based on intention to move the paralyzed arm is considered beneficial, as the system performs the movements of the paralyzed side. Therefore, we developed a task-based operating function for moving, gripping, and releasing the system using voice information that enables detailed movement instructions and BES that reflect the intention to move the paralyzed arm as an operating method that does not require the unaffected arm. The force during the gripping operation is taught and set at the start of the work support teaching. This force is learned using the previously developed gripping force teaching function (Toyama et al., 2017). The details of this function will be provided in Section 2.3.4.2.

In the voice operation, the desired operation’s content is input directly, enabling accurate and detailed operation. The system also has a set of format input rules, such as “move 10 cm forward,” which require input of a specific movement amount and direction, accepting only inputs that conform to these rules and thus preventing actions that result from incorrect input or misrecognition. In addition, commands corresponding to expected movement requests, such as “Come in front of me,” are provided. During voice recognition, the LED blinks gradually, and the system notifies the user with a voice message when recognition fails or when an unregistered command is input. In the operation using BES, the system can be operated according to the user’s own intention to move by connecting the system with the movement intentions from the brain–nervous system. This enables intervention in the motion of a task-based robot hand-arm according to the user’s intention information based on the BES and facilitates a more intuitive operation. In this study, we initially assumed that the system would be applied to patients with severe paralysis who can speak and determine the use of voice information to specify detailed tasks and operations. The use of the BES in the paralyzed arm was adapted depending on the patient. Even if the signal is weak, the part of the signal reflecting voluntary movement can be used to operate a corresponding part of the system; for example, the signal in the flexion/extension of the fingers will be used to trigger the gripping/releasing operation of the system. Even in cases where operation by a BES is difficult, it is possible to perform all operations using voice commands.

#### 2.3.2 Division of support actions into movement phases

The series of support actions is divided into individual movement phases based on the three phases of moving, gripping, and releasing by separating them into different types of movement. For the “move” phase, the following rules were added to the division, considering the characteristics of the system operation:

(A) Owing to the characteristics of the voice-operated function developed in this study, only one direction of movement can be indicated at a time; therefore, the final position and posture are more crucial than the movement path. Thus, the continuous part of the moving action is divided into a single movement phase, where the final position and posture are set as the target position and posture. In work support based on the learned data, movement to the target position and posture is executed using the shortest path that considers the system’s range of motion.
(B) Even when the moving action is continuous, there may be situations where the robot is required to stop at a certain position. Therefore, when the user’s unaffected arm interacts with the object gripped by the system, the user is prompted to input a stop command: “Keep it.” When a stop command is input, the movement is divided into phases before and after the stop, even if the movement type before and after the stop is “Move.”

The specific motion information for each movement phase to be learned is as follows: for movement, the target position and orientation of the hand; for gripping, the type of grip (two- or four-finger) and the grip force value; for releasing, the hand opening with full finger extension. Since the movements and positional relationships that facilitate the same task are generally consistent, the system provides identical support actions for each task. This consistency allows the user to easily anticipate the movements, contributing to smooth coordination. Furthermore, since the tasks are performed together with the user’s unaffected arm, slight misalignments can be compensated for by the unaffected arm. The important point is that these support actions during the movement phases are performed according to the user’s intentions, such as the intention to move the paralyzed arm and the unaffected arm.

#### 2.3.3 Phase-shift condition learning

Regarding the BES of the paralyzed arm, a signal that reflects the user’s intention is used depending on the degree of paralysis. Therefore, as a default learning, we developed a learning function for the phase-shift conditions mainly based on the movement information of the unaffected arm and voice so that it can be applied regardless of the user. For user-tailored learning, we developed a learning function that allows the BES of the paralyzed arm to correspond to phase-shift conditions in combination with or in place of the default learning conditions. In actual operation, the degree to which a signal reflecting intention can be obtained from the BES of the paralyzed arm is first checked, and the information used for the phase-shift condition is then determined according to the user.

##### 2.3.3.1 Default learning

In default learning, phase-shift conditions primarily rely on sensor information from the movement of the unaffected arm, such as the distance data DS between the robot hand and the work object and the COF on the surface of the robot finger. DS reflects the movement of the unaffected arm, bringing the work object closer to or away from the system’s hand. COF indicates the direction in which force is applied by the unaffected arm to the object gripped by the system. The system compares sensor information before and after each movement phase, learning phase-shift conditions based on the changes in sensor values. The changes in DS and COF before and after the start of the movement phase—ΔDS and ΔCOF—are calculated and determined using Equations 1 and 2, respectively.

$$∆DS=\left\{\begin{array}{l} {DS}_{phase\_shift}-{DS}_{hold}\;\left(\left|{DS}_{phase\_shift}-{DS}_{hold}\right|>{∆DS}_{threshold}\right).\\ 0\;\left(else\right)\; \end{array}\right.$$ (1)

$$∆COF=\left\{\begin{array}{l} {COF}_{phase\_shift}-{COF}_{hold}\;\left(\left|{COF}_{phase\_shift}-{COF}_{hold}\right|>{∆COF}_{threshold}\right).\\ 0\;\left(else\right)\; \end{array}\right.$$ (2)

**FIGURE 3:**
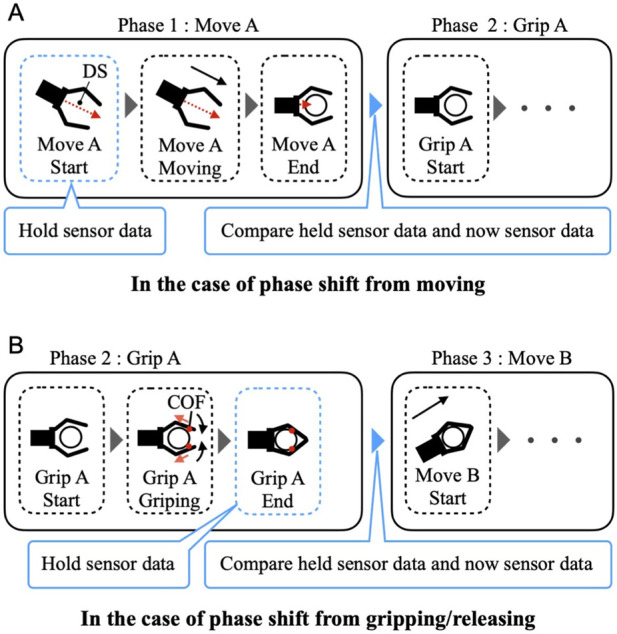
The timing of sensor values is held for phase-shift condition learning. **(A)**In the case of phase shift from moving, the sensor values are held at the start of movement. **(B)**In the case of phase shift from gripping/releasing, the sensor values are held when the action is completed.

Here, *DS*_*phase_shift*_ and *COF*_*phase_shift*_ are the values measured during the phase shift, and *DS*_*hold*_ and *COF*_*hold*_ are the values measured and held before it. *ΔDS*_*threshold*_ and *ΔCOF*_*threshold*_ are thresholds to determine if each sensor value has changed and is larger than the measurement noise of each sensor. The hold for each sensor value is based on the following rules:

(A) In the “move” phase, each sensor value is held at the start of the movement because a transition condition to the next phase may occur at the completion of the movement; for example, if there is an object within the grip range after the movement and the DS value is decreasing (Figure 3A).
(B) In the “grip“/”release” phase, it is assumed that the contact position between the robot finger surface and the gripped object changes during the grip/release movement of the system, leading to potential changes in the COF. In this study, to capture the changes in the COF caused by the user’s unaffected arm movement, we removed the component of the COF change caused by the system movement by holding each sensor value at the completion of the grip/release movement (Figure 3B).

**FIGURE 4:**
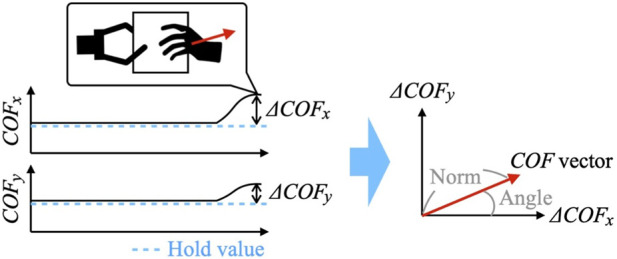
COF vector with the COF hold value as the origin.

The ΔCOF is captured as a COF vector (*ΔCOF*_*x*_,*ΔCOF*_*y*_) with the COF hold-value as the origin (Figure 4). The COFs of a pair of facing fingers were used to learn the phase-shift condition. This is because, in both cases of two- and four-finger gripping, measuring the COFs in a pair of two fingers enables the system to capture the direction in which the unaffected arm is applying force to the gripped object. For two-finger gripping, the system utilized the COF values from tactile force sensors mounted on the fingers to learn the phase-shift conditions. Similarly, for four-finger gripping, the COF values from tactile force sensors on the upper two fingers were used for learning.

Since there is assumed to be a certain degree of variability in the movement of the unaffected arm, which acts as the phase-shift condition, a value that allows a certain degree of error in the magnitude of the change for ΔDS and in the norm and direction for the COF vector is learned as the phase-shift condition; additionally, if ΔDS and ΔCOF for all sensors at the phase shift are 0—no change observed in any sensor value—the voice command “next” is learned as a phase-shift condition. During the work support after learning, when the phase-shift condition is based on a voice command, the LED ring at the base of the system turns orange to notify the user. Furthermore, the first and last movement phases of the work support involve movement from the standby position (home position) and returning to it, respectively. Therefore, the first movement phase is learned to automatically phase shift when the work support starts, and the last movement phase is learned when the previous movement phase is completed. This process enables automatic learning of phase-shift conditions from sensor information on human intention and movement obtained during work.

##### 2.3.3.2 User-specific learning

In default learning, the phase-shift condition to the movement phase is mainly based on sensor information regarding the motion of the unaffected arm. Additionally, for users whose BES reflects their movement intention and can be measured in their paralyzed arm, the framework allows the user to adapt the BES to the phase-shift conditions, either in combination with or by replacing the default learning conditions. If the BES is difficult to detect owing to paralysis, it cannot be used for detailed operations, such as moving the system’s hand position. However, by applying the intention information from the paralyzed arm’s BES to the phase-shift conditions for the movement phases that constitute learned work support, cooperative work based mainly on the paralyzed arm’s movement intention becomes possible.

#### 2.3.4 Other function details

##### 2.3.4.1 Detection of intention to move from the paralyzed arm’s BES

**FIGURE 5:**
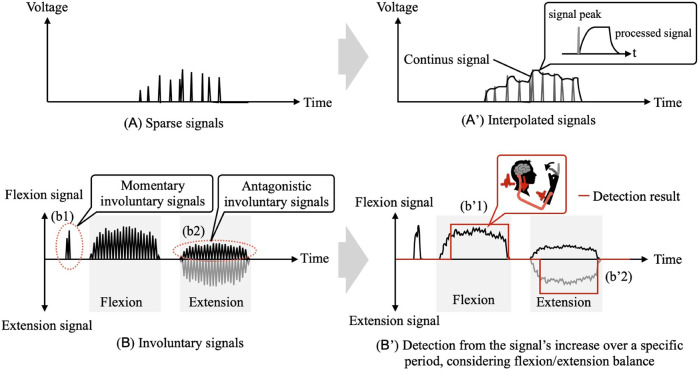
**(A,B)** Expected BES of the paralyzed arm. **(A',B')** Detection method for intention to move based on the BES using the developed system.

In this study, we focus on patients with severe paralysis, detecting and utilizing the intention of flexion/extension movements from areas where signals corresponding to their intention can be confirmed. In the BES of the paralyzed arm, it is expected that, in addition to weak signals, 1) sparse signals (Figure 5A) and 2) sudden involuntary signals (Figure 5(b1)) as well as signals from the antagonistic muscles (Figure 5(b2)) will be present (Raghavan, 2015).

To address 1), a first-order lag system-based filter, which uses the signal peak as input, is utilized to interpolate the sparse signals into continuous signals while preserving the characteristics of the voluntary signals (Figure 5A') (Masahiro et al., 2009). The intention detection considers the involuntary signals mentioned in 2). First, as a response to the involuntary signals from the antagonistic muscles, a candidate intention, *FE*_*candidate*_, is determined by considering the balance of flexion/extension signals using Equation 3.

$${FE}_{candidate}=\left\{\begin{array}{l} \prime \prime Flexion^{\prime \prime }\left(V_{flexion}\;>\;F_{threshold}\;\cap \;G_{flexion}V_{flexion}\;>\;G_{extension}V_{extension}\right)\\ \prime \prime Extension^{\prime \prime }\left(V_{extension}>E_{threshold}\cap G_{flexion}V_{flexion}<G_{extension}V_{extension}\right).\\ \prime \prime {None}^{\prime \prime }\left(else\right) \end{array}\right.$$ (3)

*V*_*flexion/extension*_ represents the signals from the flexor/extensor muscles, which have been interpolated into continuous signals. *F/E*_*threshold*_ is the threshold for detecting a signal increase that reflects the intention to move, and *G*_*flexion/extension*_ is the gain for balance adjustment. Then, as a response to the sudden involuntary signals, the final estimation of flexion/extension intention is determined based on sustained intentions derived from *FE*_*candidate*_ over a specific period using Equation 4.

$${FE}_{estimatied}=\left\{\begin{array}{l} \prime \prime Flexion^{\prime \prime }\;\left(F_{time}>T_{threshold}\right)\\ \prime \prime Extension^{\prime \prime }\;\left(E_{time}>T_{threshold}\right).\\ \prime \prime None^{\prime \prime }\;\left(else\right)\; \end{array}\right.$$ (4)

Here, *F*_*time*_ and *E*_*time*_ represent the time during which flexion and extension were continuously estimated in *FE*_*candidate*_, respectively, and *T*_*threshold*_ is the temporal threshold for the final estimation of flexion/extension intention. Each parameter, including *F/E*_*threshold*_ and *G*_*flexion/extension*_, is adjusted for each patient during the initial application. Through the above process, detection of intention to move based on the BES from the paralyzed arm (Figure 5B') is achieved.

##### 2.3.4.2 Grip force learning and control function

**FIGURE 6:**
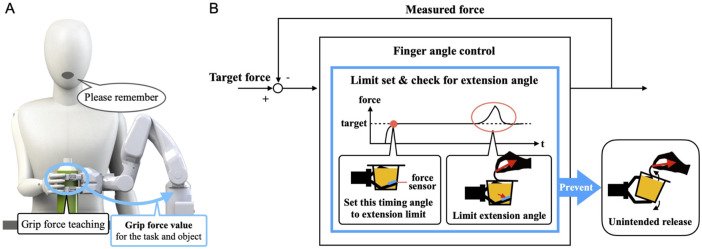
**(A)** State of grip force teaching and learning. **(B)** Overview of the developed grip force control for the cooperative work.

The gripping force is learned according to the task and object using the previously developed gripping force teaching function (Toyama et al., 2017) (Figure 6A). First, the system’s hand is moved to a position which makes it easier to teach the gripping force using voice commands. Then, when the user grips the object from above the system’s hand and says “Remember,” the system memorizes the sensor value measured by the tactile force sensor for the task and object.

When the system grips an object, and the user’s unaffected arm is involved, such as turning over the lid of a container, the object is temporarily pressed against the system’s finger surface depending on the direction in which the lid is turned over, increasing the contact pressure on the system’s finger surface. In such cases, a simple grip force control that keeps the force constant causes the fingers to open to reduce the force or fail to maintain the balance of forces, leading to the gripped object being dropped. To address this issue, in addition to the normal grip force control, a control system was added that memorizes the finger movement angle when the target grip force is first reached and limits the extension movement angle based on the memorized angle (Figure 6B). This enables the system to prevent unintended release of the gripped object during cooperative work because the motion angle at which the object is gripped is maintained even when the work object is temporarily pressed against the system’s finger surface by the motion of the unaffected arm. In this case, the force applied to the work object temporarily increases; however, it is considered an increase in the force necessary to perform the work, such as when the lid of a container is opened by turning it.

## 3 Experiments and results

To confirm the feasibility and usefulness of a cybernic robot hand-arm in assisting the work of patients with a single upper-limb dysfunction, we conducted a pilot study involving actual patients. The study was carried out in accordance with the experimental protocol approved by the ethics committee of CYBERDYNE, Inc., where the experiments took place. Prior to the experiment, all participants were briefed about the study and their informed consent was obtained. In Section 3.1, we confirmed the feasibility of the proposed method for learning cooperative work support with the unaffected arm and providing work support based on the learned data with the cooperation of three patients. To learn the phase-shift conditions, we applied a default learning method that primarily utilized the motion information of the unaffected arm, which is applicable regardless of the degree of paralysis in the user’s arm. The BES of the paralyzed arm was used as operational input to teach the support movements, if available. In Section 3.2, we confirmed the feasibility of work support based on learned data when a phase-shift condition using the BES of the paralyzed arm was added as user-specific learning. This experiment involved a patient for whom the BES of the paralyzed arm was available. Finally, in Section 3.3, as an effort to support daily tasks, we applied the system to various daily tasks that a patient had difficulty performing, with the cooperation of that patient. We aimed to determine whether the system is capable of supporting a range of tasks encountered in daily life.

### 3.1 Experiment on learning and implementation of work support in cooperation with the unaffected arm

#### 3.1.1 Design of the experiment

To confirm that the proposed system can learn work support in cooperation with the unaffected arm and provide work support based on the learned data, we conducted experiments involving upper-limb work support for an individual with paralysis in one arm. The tasks were “opening a medicine package,” “opening a PET bottle,” and “eating a jelly cup,” which are difficult for individuals with a single upper-limb dysfunction because they require both hands (Sköld et al., 2004; Mancuso et al., 2015). To maintain a constant task difficulty level, participants received specific instructions regarding the task details, such as tilting the jelly cup as its contents decreased. As preparation for the experiment, the grip force for each task was taught using the grip force learning function (Toyama et al., 2017), which was developed and confirmed for its basic performance. Additionally, the BES of finger flexion/extension from the paralyzed arm were measured and utilized to trigger the grip/release operation of the robot hand if it was available as a signal corresponding to the intended movements. When it was difficult to use the signal as an intention signal, voice was used for all operations.

In the experiment, we initially confirmed that the participant could operate the system using intention information via voice and the BES of the paralyzed arm to execute the target task while teaching support actions. We also confirmed that the system could learn these support actions and phase-shift conditions corresponding to the user and the task using the default learning method. Subsequently, we confirmed that the system could cooperate with the unaffected arm to perform the target task by providing work support based on the learned data. The work support based on the learned data was implemented three times for each task. Lastly, we conducted a subjective evaluation of the system and its performance using a questionnaire. The questionnaire details, along with the results, are described in the following section.

#### 3.1.2 Participants

The experiment was conducted with three patients with paralysis of one upper limb.

Participant A was a 27-year-old man with severe paralysis of the left upper limb due to a brachial plexus injury. Although he could slightly move his left-hand fingers, he was unable to use them for functional tasks. The BES in finger flexion/extension of the left upper limb exhibited a weak and sparse signal; however, it corresponded to his intention, confirming the system’s ability to detect his intention to move, thus enabling the operation of the system. Consequently, the BES in the finger flexion/extension of the left upper limb was utilized as a trigger for the grip/release operation of the robot hand, while voice was employed for other operations.

Participant B, a 56-year-old man, experienced right hemiplegia due to the aftereffects of cerebral hemorrhage, resulting in severe paralysis of the right upper limb. Despite attempts, he was unable to move his right upper limb. Additionally, he reported a slight slurring of the tongue following the illness. No signal corresponding to intention was detected in the BES of the right-hand fingers during flexion/extension. It was confirmed that the participant could execute all system operations using his voice. Consequently, voice was employed for all operations.

Participant C, a 68-year-old man, had right hemiplegia due to cerebral infarction, resulting in right upper limb paralysis. Although he could move his right upper limb, his grip strength was weak, and he experienced difficulty in movement. He also reported approximately 20% lower speech ability compared to his normal state. The BES in the finger flexion/extension of his right upper limb exhibited a signal corresponding to his intention, confirming the system’s ability to detect his intention to move and thus enabling the operation of the system. Additionally, it was confirmed that the participant could operate the system using voice. Consequently, the BES in finger flexion/extension of the right upper limb was utilized as a trigger for grip/release operation of the robot hand, while voice was employed for other operations.

Participants B and C had scheduled rehabilitation sessions before or after the experiment. To minimize their burden, the experiment was divided into 3 days, with each day focusing on one task.

#### 3.1.3 Results

##### 3.1.3.1 Learning of work support

It was confirmed that each participant could successfully complete the target task while operating the system and teaching support actions using intention information from voice and the BES of the paralyzed arm. Additionally, it was confirmed that the system could learn support actions and phase-shift conditions corresponding to the participants and tasks. An example from Participant A is provided below.

**FIGURE 7:**
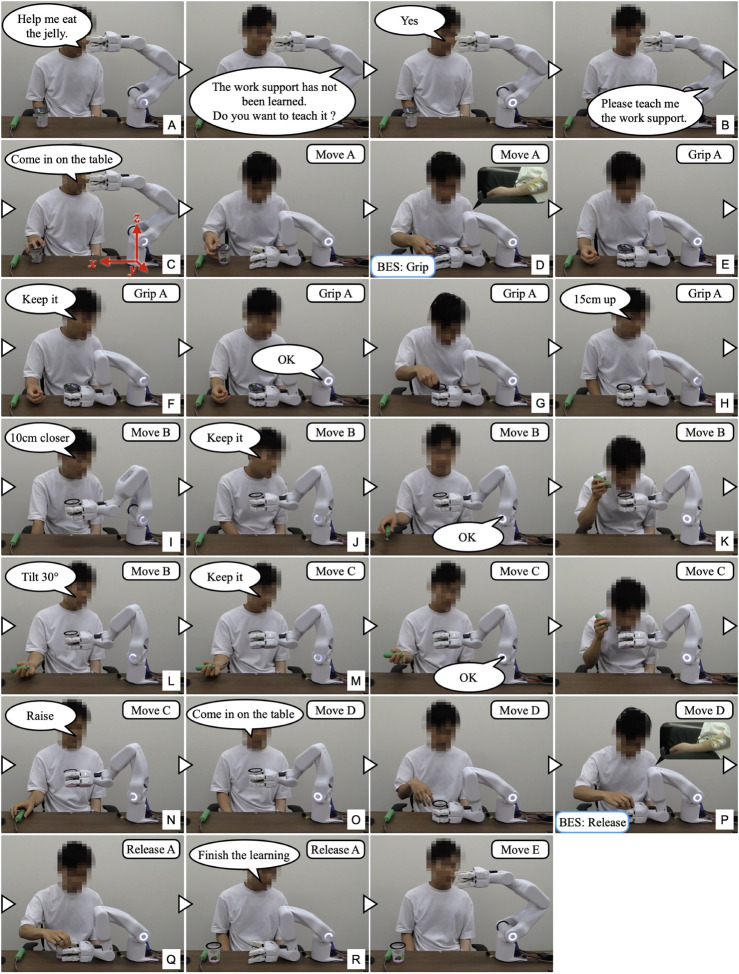
The work support learning the “eating a jelly cup” task. **(A)** Participant inputs task name; **(B)** System prompts for support action teaching; **(C)** Participant instructs to move hand to open work position; **(D,E)** Intention to flex fingers is detected by BES of paralyzed arm, and system grips jelly cup; **(F)** Participant instructs to hold cup; **(G)** Cup is opened with unaffected arm; **(H,I)** Participant instructs to move cup close to mouth; **(J)** Participant instructs to hold cup in position; **(K)** Participant eats jelly; **(L,M)** Participant instructs to tilt cup and hold it in position as it nears emptiness; **(N,O)** Upon finishing jelly, participant instructs to move cup to position for easier receiving; **(P,Q)** Intention to extend fingers is detected by BES, and system releases cup; **(R)** Participant instructs to finish learning.

Figure 7 shows the experiment for the “eating a jelly cup” task. First, the participant verbally input the name of the task for which he wanted work support (Figure 7A). As work support had not been learned, the system prompted the participant to teach the support action (Figure 7B). Next, the participant instructed the system to move its hand to the open work position, which the system executed (Figure 7C). The unaffected arm then brought the jelly cup into the gripping range of the system. Subsequently, the intention to flex the fingers was detected by the BES of the paralyzed arm (Figure 7D), and the system performed the gripping action. Adjusting to the size and position of the jelly cup, the system’s four fingers gripped it firmly (Figure 7E). The participant then instructed the system to hold the jelly cup in that position (Figure 7F) and opened it using the unaffected arm (Figure 7G). Following that, the participant instructed the system to move the jelly cup close to his mouth to facilitate eating, which the system executed (Figures 7H, I). Afterward, the participant instructed the system to hold the jelly cup in position (Figure 7J) and ate the jelly using a spoon with the unaffected arm (Figure 7K). As the jelly cup approached emptiness, the participant instructed the system to tilt the cup and hold it in that position (Figures 7L, M). Upon finishing the jelly, the participant instructed the system to move the cup to a position where it would be easier to receive it (Figures 7N, O). As the unaffected arm received the cup, the intention to extend the fingers was detected from the BES of the paralyzed arm (Figure 7P), and the system released the cup (Figure 7Q). After the participant received the cup, the system was instructed to complete the learning (Figure 7R); the system’s hand returned to its initial position, completing the work support and learning process.

**FIGURE 8:**
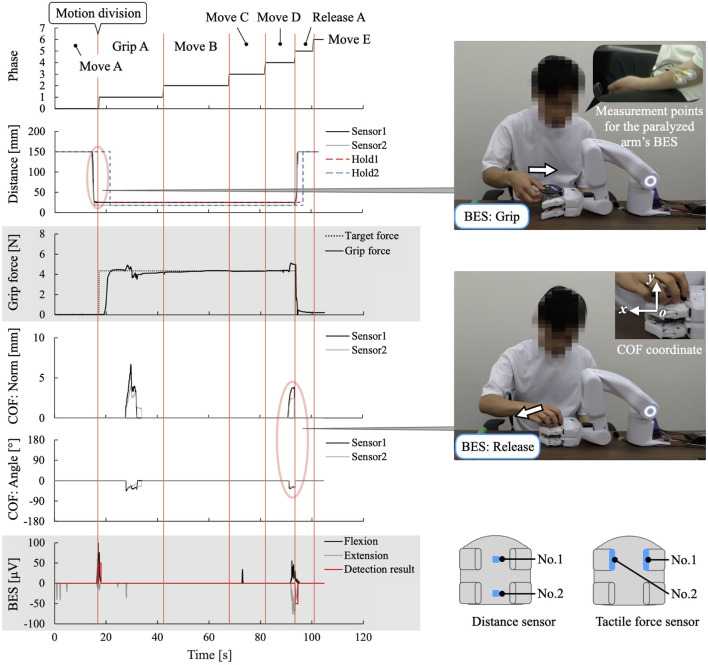
Sensor data measured in learning the “eating a jelly cup” task. The red circles show the characteristic changes in sensor values observed when the next action occurs.

Figure 8 shows the sensor data measured during the experiment, and Table 1 lists the learned data obtained. From this learning, the taught sequence of support actions was divided into seven phases: Move A, Grip A, Move B, Move C, Move D, Release A, and Move E. The phase-shift conditions for the first and last phases, Phases 0 (Move A) and 6 (Move E), respectively, were learned to automatically perform the phase shifts according to the developed learning algorithm. For the phase-shift condition to Phase 1 (Grip A), the movement of the unaffected arm carrying the jelly cup into the gripping range of the system was learned as a decrease in the distance to the working object at distance sensors 1 and 2. For the phase-shift conditions to Phases 2 (Move B), 3 (Move C), and 4 (Move D), voice commands were learned as conditions because no change was detected in the sensor information before and after the phase shift. For the phase-shift condition to Phase 5 (Release A), the force exerted by the unaffected arm in the direction of receiving the jelly cup was learned as the movement of the COF measured on the surface of the robot finger gripping the jelly cup.

**TABLE 1:** Learning result of the “eating a jelly cup” task with participant A.

Phase	Movement		Phase-shift condition
0	Move A Position (350, 0, 30) [mm] Posture (0, 90, 0) [deg]	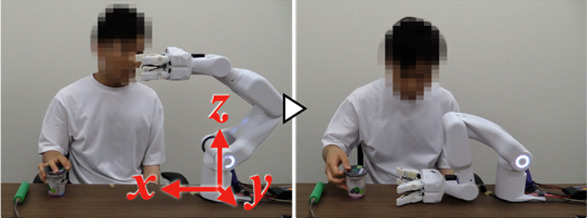	Automatic
1	Grip A: 4.4 [N]	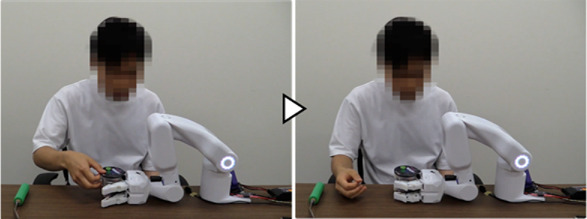	ΔDistance1 ≦ −112 [mm] ΔDistance2 ≦ −119 [mm]
2	Move B Position (350, −100, 180) [mm] Posture (0, 90, 0) [deg]	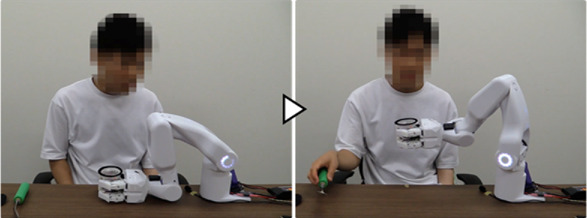	Voice command
3	Move C Position (350, −100, 180) [mm] Posture (0, 90, 30) [deg]	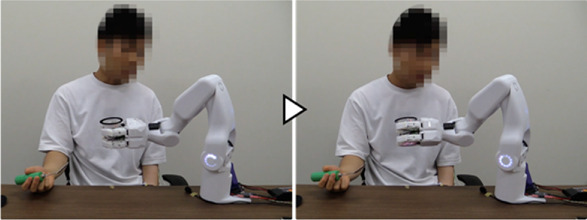	Voice command
4	Move D Position (350, 0, 30) [mm] Posture (0, 90, 0) [deg]	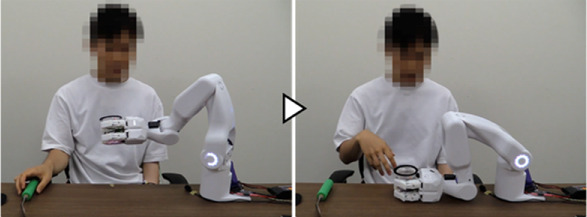	Voice command
5	Release A	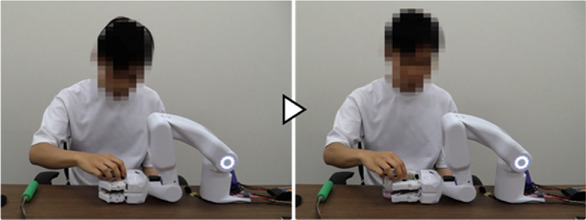	Norm1 ≧ 3.1 [mm] Norm2 ≧ 2.0 [mm] −57 ≦ Angle1 ≦ 3 [deg] −62 ≦ Angle2 ≦ −2 [deg]
6	Move E Position (250, 0, 400) [mm] Posture (0, 90, 00) [deg]	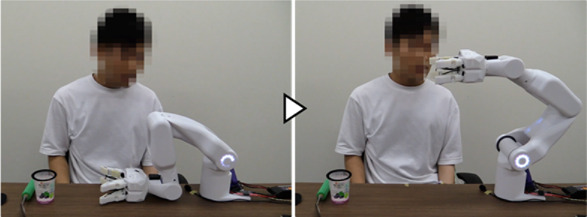	Automatic

Position (x, y, z), Posture (Roll, Pitch, Yaw)

##### 3.1.3.2 Work support based on the learned data

It was confirmed for each participant that the system executed each support action in response to and in conjunction with the work phase and the movement of the unaffected arm. Phase shifts to each movement phase were based on the learned data and sensor information, ensuring that the participant could complete the target task. All participants were able to successfully complete all three trials for each task. Table 2 illustrates a comparison of work time with and without teaching support. Work time significantly decreased with the implementation of work support based on learned data. An example from Participant A is provided below.

**TABLE 2:** Comparison of work time.

	Participant A		Participant B		Participant C	
	WT [s]	WLD [s]	WT	WLD	WT	WLD
Opening a medicine package	48	26 ± 2.2 (46%↓)	96	36 ± 4.5 (63%↓)	70	39 ± 5 (44%↓)
Opening a PET bottle	51	29 ± 0.5 (44%↓)	66	27 ± 1.7 (60%↓)	74	34 ± 2.1 (55%↓)
Eating a jelly cup	104	59 ± 1.7 (44%↓)	152	63 ± 2.5 (58%↓)	126	80 ± 0.9 (36%↓)

WT: Work time with teaching WLD: Work time with learned data, represented as mean ± standard deviation Numbers in parentheses indicate the reduction in time when comparing the mean value of WLD to WT

**FIGURE 9:**
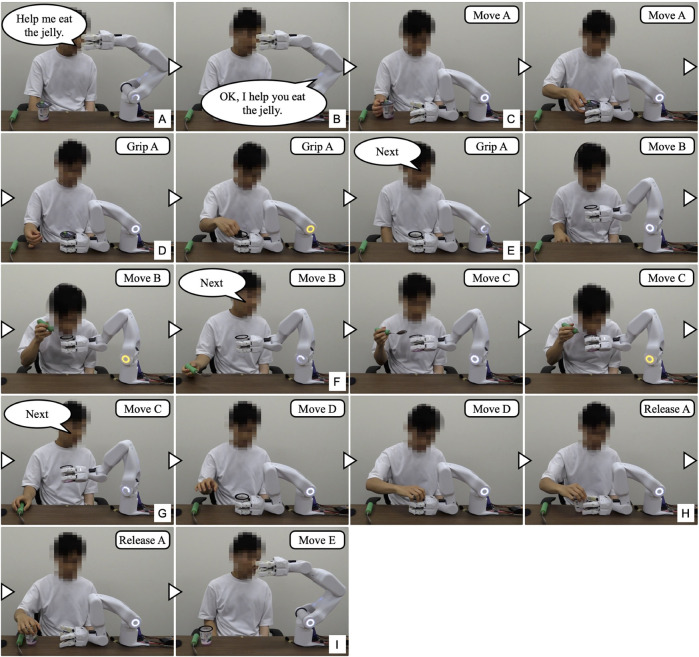
The work support of the “eating a jelly cup” task based on the learned data. **(A)** Participant inputs task name; **(B)** Work support for target task is initiated; **(C)** Move A is performed automatically; **(D)** Grip A is performed when cup is brought into system’s hand; **(E–G)** Move B, Move C, and Move D are triggered by voice command “Next”; **(H)** Release A is performed as participant attempts to receive cup with unaffected arm; **(I)** Move E is performed automatically.

**FIGURE 10:**
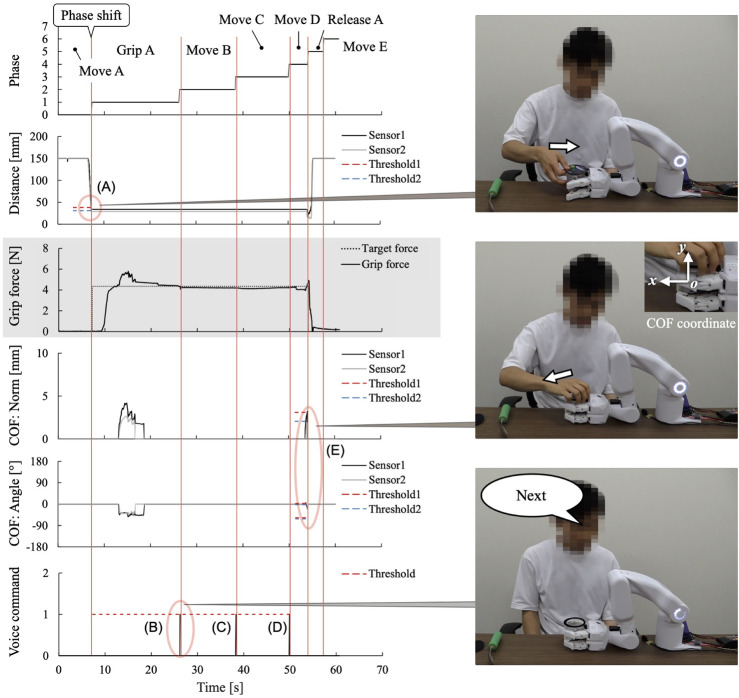
Sensor data measured in the work support of the “eating a jelly cup” task based on the learned data. **(A)** Phase shift to Phase 1 (Grip A) occurs when jelly cup is brought into system’s hand and distance values fall below a threshold set based on learned data. **(B–D)** Phase shifts to Phases 2 (Move B), 3 (Move C), and 4 (Move D) are triggered by voice command “Next”; **(E)** Phase shift to Phase 5 (Release A) occurs as participant attempts to receive cup with unaffected arm; the norm of the COF vector due to COF movement exceeds the threshold, and the angle is within the threshold.

Figure 9 shows the work support of the “eating a jelly cup” task based on the learned data shown in Table 1, and Figure 10 shows the sensor data measured during the work. First, the participant entered the name of the task for which he wanted work support through voice input (Figure 9A). As work support for the target task had already been learned, it was initiated (Figure 9B). Phase 0 (Move A) was performed automatically (Figure 9C), and the phase shift to Phase 1 (Grip A) was performed when the jelly cup was brought into the gripping range of the system by the unaffected arm and the distance to the work object in the two distance sensors fell below a threshold value set based on learned data (Figures 9D, 10A). Phase shifts to Phases 2 (Move B), 3 (Move C), and 4 (Move D) were triggered when the voice command “Next” was input at the intended timing by the participant (Figures 9E–G, 10B–D. The participant confirmed the voice command’s effect by observing the orange illumination of the system’s LED. Phase shift to Phase 5 (Release A) occurred when the participant attempted to receive the jelly cup with the unaffected arm; the norm of the COF vector due to the COF movement exceeded the threshold, and the angle was within the threshold (Figures 9H, 10E). Finally, a phase shift to Phase 6 (move E) was automatically performed (Figure 9I). Thus, each support action corresponded and linked with the work phase and movement of the unaffected arm by phase shifting to each movement phase based on learned data and sensor information, enabling the completion of the target work.

##### 3.1.3.3 Subjective evaluation by questionnaire

Table 3 presents the results of a questionnaire administered after the experiment. Each item is rated on a 5-point scale, with “1” indicating strongly disagree and “5” indicating strongly agree. Participant B provided detailed answers, including decimal points for some of the evaluation items, which were used in the evaluation. The questionnaire survey confirmed that the system’s operation and work time were generally highly evaluated in the work-support learning experiment. Additionally, for the work support experiment based on the learned data, high overall evaluations were obtained for both work support and time. Furthermore, in both experiments, participants A and C, who used BES for operation during learning, experienced the system as if it were their own arm.

**TABLE 3:** Questionnaire results of the experiment by participants. (five-grade evaluation).

Questionnaire	Participant A	Participant B			Participant C		
	M, P, J	M	P	J	P	M	J
1. About work while teaching work support to the robot							
1.1 Were you able to operate the robot as intended?	5	4	4	5	5	5	5
1.2 Do you think the working time is within the range that you can do in your daily life?	5	3	5	5	5	5	5
1.3 Did you feel that the robot was like your arm?	5	1	1	1	5	5	5
2. About work after teaching							
2.1 Did the robot help you with the work as you intended?	5	3	5	3	5	5	5
2.2 Do you think the working time is within the range that you can do in your daily life?	5	3.5	5	4	5	5	5
2.3 Did you feel that the robot was like your arm?	5	1.5	1	1	5	5	5

M: opening a medicine package, P: opening a PET bottle, J: eating a jelly cup

### 3.2 Experiment using a voluntary control system in which the phase-shift function using BES is added to the control system after learning

When the BES are difficult to detect owing to paralysis, it is difficult to use them for detailed operations such as adjusting the system’s hand position, even though it is possible to estimate movement intentions at a basic level, such as flexion/extension and weakness/non-weakness. However, for the movement phases constituting learned work support, intention information from the paralyzed arm’s BES regarding movement intentions, such as moving, gripping, and releasing, can be utilized as the trigger for phase shifts, even at a basic level. This allows for the provision of work support based on the movement intentions of the paralyzed arm. Therefore, we conducted a work support experiment where the BES was incorporated into the phase-shift conditions for the learned data to confirm that phase shifts could be performed according to the movement intention based on the BES of the paralyzed arm and that work support could be provided in cooperation with the unaffected arm according to the user’s intention.

The experiment was conducted with the cooperation of participant A, who had paralysis in his left upper limb due to nervous system damage (see Section 3.1.2). In the subjective evaluation of the system and work, questionnaire items related to work support based on learned data were selected from the evaluation items used in the experiment described in Section 3.1. The learned data for the work support of “eating a jelly cup” obtained in the previous experiment (Section 3.1) was utilized for this experiment. For movement phases (Phases 2, 3, and 4), where voice commands were initially learned as phase-shift conditions, the BES reflecting the intention to move the paralyzed hand-arm position were used instead. These captured the intention to move the fingers and wrist, and the motion intention of flexion/extension was used as a phase-shift condition. This decision was based on observations from preliminary measurement tests, where small, complex movements of the fingers and wrists were observed when the participant was consciously attempting to move the hand position of the paralyzed arm. Owing to the overlapping muscle groups responsible for each movement, the same electrode was used for measurement. The work support was implemented three times.

**FIGURE 11:**
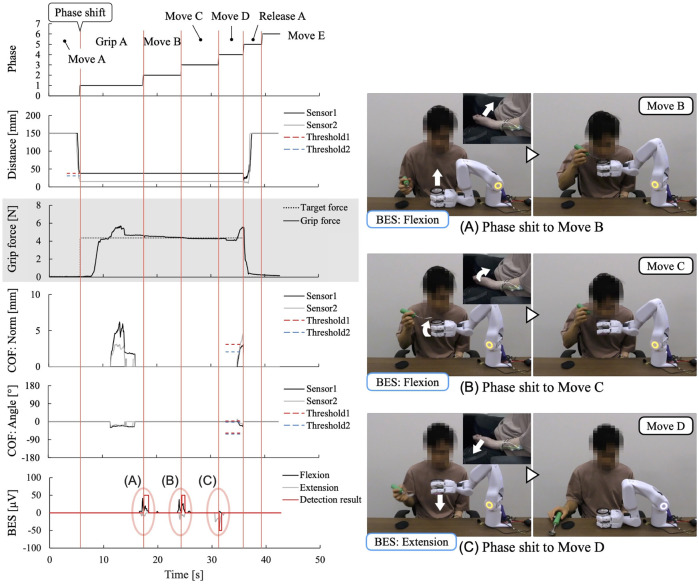
Measured sensor data in the work support with a control system with BES added as phase-shift conditions instead of the learned conditions(voice command). **(A–C)** Phase shifts based on BES of paralyzed arm.

Figure 11 shows the experiment and measured sensor data. Each support action was performed in response to and in conjunction with the work phase and movement of the unaffected arm, resulting in the completion of the target task in all trials. As shown in Figures 11A–C, phase shifts to the movement phases with phase-shift conditions based on the BES were performed when the intention of flexion/extension was detected from the BES of the fingers/wrist, reflecting the intention to move the hand position of the paralyzed arm. For the BES of the paralyzed arm, the phase-shift condition was the intention to flex or extend the fingers and wrist. However, focusing on the motion intention of the wrist, it was observed that the motion intention based on the BES during the phase shift corresponded to the detailed movement content of the system. During the movement to bring the gripped jelly cup close to the mouth, the intention to flex the wrist to bring the paralyzed arm’s hand close to the mouth was detected (Figure 11A). During the wrist-tilting movement, the intention to flex the wrist of the paralyzed arm was detected (Figure 11B). When the jelly cup was lowered onto the desk, the intention to extend the wrist to lower the paralyzed arm’s hand downward was detected (Figure 11C). The average work time was 59 s, with a standard deviation of 1.7 s before changing the phase-shift conditions from voice commands to BES; however, in this experiment, it was significantly reduced to 44 s, with a standard deviation of 0.9 s.

The results of the questionnaire after the experiment were as follows. “Did the robot help you with the work as you expected?” received a score of 5, “Do you think the working time is within the range that you can do in your daily life?” received a score of 5, and “Did you feel that the robot was like your arm?” also received a score of 5. High scores were confirmed for work support and time. Furthermore, the participant felt that the system was similar to his arm. Additionally, the participant commented, “I felt as if the robot was my arm more than in any other experiment I have done so far.”.

### 3.3 Effort to support daily tasks

**FIGURE 12:**
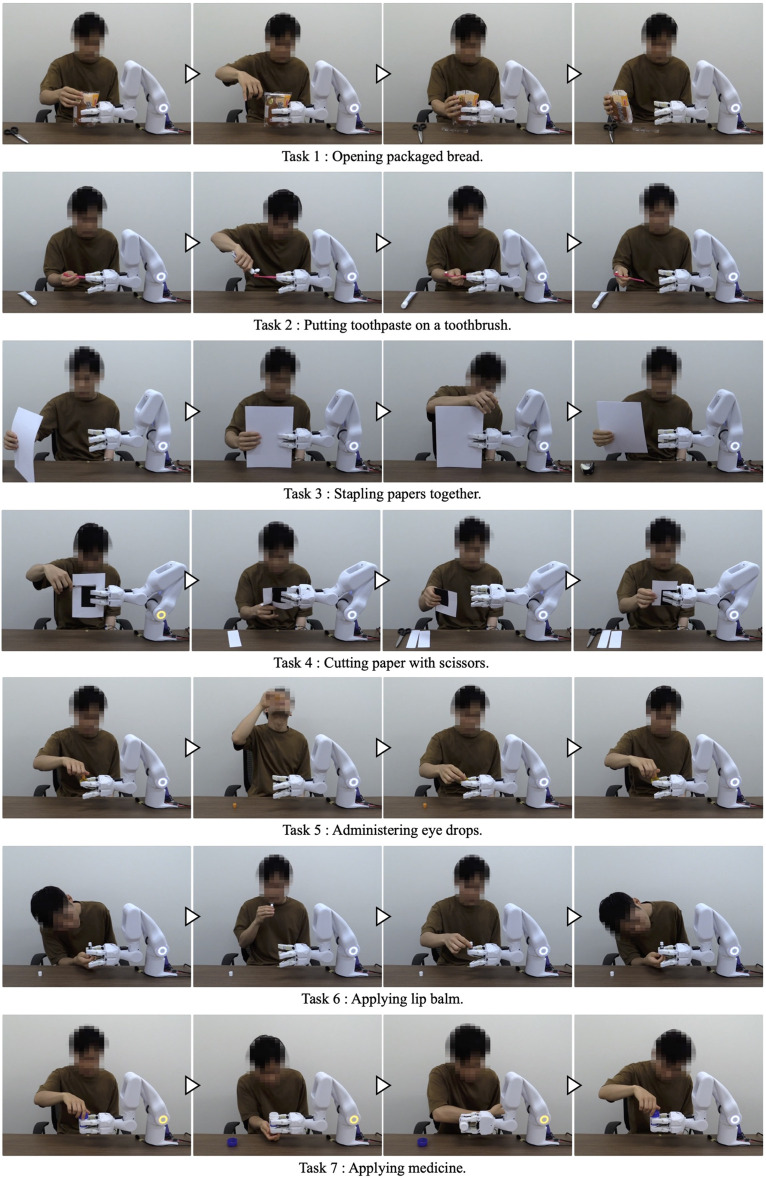
Work support based on the learned data for target tasks 1 to 7.

By applying the system to various tasks with which the participant had difficulties in daily life, in addition to the three tasks targeted in Section 3.1, we confirmed that the system could support a variety of tasks in the users’ daily lives. The experiment was conducted with Participant A’s cooperation. The following seven tasks were deemed safe to perform with the system from among the tasks with which the participant actually had difficulties in daily life: 1) opening packaged bread, 2) putting toothpaste on a toothbrush, 3) stapling papers together, 4) cutting paper with scissors, 5) administering eye drops, 6) applying lip balm, and 7) applying medicine. The system was applied to these tasks, and it was confirmed that each task could be performed (Figure 12). Considering the burden on the participant due to the large number of tasks, the work support after learning was conducted once for each task.

## 4 Discussion

Patients with a single upper-limb dysfunction face various challenges, such as difficulty or impossibility in performing tasks and overloading on one arm owing to their inability to use the other arm. A robot hand-arm to replace the paralyzed limb would be beneficial in addressing this fundamental problem. However, conventional robot hand-arms for persons with upper-limb dysfunctions have been designed for individuals with bilateral upper-limb dysfunction, and tasks are performed only by the robot hand-arm. None have been designed to perform cooperative tasks with the unaffected arm of individuals with a single upper-limb dysfunction. In conventional methods, there have been issues such as restricting the physical movements necessary for cooperative tasks that include the unaffected arm and the inability to achieve smooth coordination with the unaffected arm in motions that reflect the user’s intentions (Figure 1). In response to these issues, this study proposed and developed a cybernic robot hand-arm that functions based on intention information, utilizing a control system that combines voluntary and autonomous control. The system autonomously executes support actions (movement phases) according to the user’s unaffected arm and the work phase, using input of BES from the paralyzed arm and the movement information of the unaffected arm as the information that reflects the intention to move in cooperative work involving both hands, as well as voice intention information. This would enable seamless cooperative work and work support that correspond to and are linked to the user’s intentions and the unaffected arm without requiring detailed operations. The system learns such work support for various tasks in daily use. Consequently, we achieved support for various upper-limb tasks for people with a single upper-limb dysfunction which were previously difficult.

### 4.1 Experiment on learning and implementation of work support in cooperation with the unaffected arm

In this experiment, we confirmed the feasibility of the proposed method for learning cooperative work support with the unaffected arm and for performing work support based on the learned data with the cooperation of three patients with a single upper-limb dysfunction, each with varying degrees of disability. To learn the phase-shift conditions, we employed a default learning method that mainly relies on motion information from the unaffected arm rather than the BES of the paralyzed arm, making it applicable regardless of the extent of paralysis. One participant was unable to generate a BES reflecting his intention but could utilize the system by using voice for all operations related to teaching support movements. These findings suggest that the system can be employed across a diverse range of patients, irrespective of the condition of their paralyzed arm.

In learning the work support, cooperative work between the system and the participant’s unaffected arm became feasible through the operation using intention information from voice and the BES of the paralyzed arm. Each participant successfully completed the target task while teaching the support action to the system. Moreover, the system learned the support actions and phase-shift conditions tailored to the participants and tasks during the actual work. Furthermore, the questionnaire (Table 3) subjectively confirmed that the intended operation could be performed and that the work time was sufficient to perform the operation in daily life. These results confirm that the participants were able to complete the target task while operating the system and teaching support actions using their intention information. Only the evaluation of work time for the “opening a medicine package” task by Participant B was lower compared to the evaluations for the other tasks. Participant B commented, “The medicines I usually take are in pill form, so I can open them with one hand. Considering this, opening them with one hand is faster.” This suggests that the lower evaluation for this task was due to comparing the work time to opening a tablet-type medicine, which can be done with one hand.

In the work support based on learned data, it was confirmed that the system executed each support action in response to and in conjunction with the work phase and the movement of the unaffected arm through phase shifts to each movement phase based on the learned data and sensor information, allowing each participant to complete the target task. The results indicated that the learned data were appropriate for cooperative work with the participant’s unaffected arm and for providing work support. Additionally, work time significantly decreased for all participants compared to before learning (Table 2). For example, in the task of opening a medicine package, all participants were able to complete it in approximately half the time after learning. This reduction is attributed to the proposed method, which features a control system combining voluntary and autonomous control and autonomously performs actions based on the input reflecting the user’s intention, allowing seamless cooperation between the system and the user’s unaffected arm without requiring detailed operations. Furthermore, the questionnaire revealed that the work support and work time were generally highly evaluated (Table 3), subjectively confirming that work support was provided according to the patient’s intention and that the work time was sufficient to perform the tasks in daily life. These results confirm that work support based on learned data enables cooperation with the unaffected arm according to the user’s intention and smoother execution of the target task. The evaluation of work time was highly rated in the questionnaire even for the work with teaching. This can be attributed to the fact that the tasks could not originally be performed with one hand, or, if possible, required a significant amount of time. This indicates that the teaching process for new tasks can be conducted in a stress-free manner and that smoother work is realized after learning. The slightly lower ratings for “opening a medicine package” and “eating a jelly cup” in Participant B compared to the other participants may be due to the following reasons. Regarding the task “opening a medicine package,” as mentioned earlier, this may have been influenced by the comparative evaluation with tablet-type medicines. For the jelly-eating task, Participant B commented, “I felt that I could not fine-tune the system’s hand position additionally, and if that was the case, I felt that the previous experiment (learning of work support) was better because I could adjust it more freely.” This comment reflects a lower evaluation compared to the previous experiment (learning of work support). Regarding the operation requests mentioned in the comment above, we aim to improve work support based on learned data so that fine adjustment of the hand position can be performed as needed.

In both the learning of work support and work support based on learned data, it was observed from the questionnaire that all the participants who used the BES for operation while learning perceived the system as a part of their body, with the highest rating of 5 (Table 3). In contrast, the participant who used only voice for operation gave a rating of 1. Thus, when the BES of the paralyzed arm is used, the system provides a sense of unity with the body. This may be because the system was connected to the user’s intention to move from the brain nervous system and performed movements in place of the paralyzed arm according to the user’s intention. It is thought that this perception of oneness with the body was maintained in work support based on learned data that did not include BES in the phase-shift condition. The phenomenon of sensory integration between the system and the user’s body is a significant discovery, and we believe that it will further strengthen the development of the field of cybernic robot hand-arms. Therefore, we aim to increase the number of participants and conduct experiments with a group that uses BES and a group that does not to verify this phenomenon. Additionally, the use of BES from the paralyzed arm provides an opportunity to move the arm that could not be used for work owing to limited motor functions caused by paralysis within its possible range of motion. Consequently, secondary effects such as the maintenance of residual functions and prevention of disuse are expected. Because it is beneficial to be able to obtain these secondary effects on a daily basis through working with the system, future research will include the verification of these secondary effects during the long-term application of the system.

### 4.2 Experiment using a voluntary control system in which the phase-shift function using BES is added to the control system after learning

In this experiment, we confirmed that phase shift based on the intention to move using the paralyzed arm’s BES and cooperative work support with the unaffected arm according to the intention were possible in the movement phase where the phase-shift condition was replaced with the BES from the default learning condition. From the results, it is inferred that even if the BES is challenging to detect and utilize for detailed operations during learning, work support aligned with the movement intention of the paralyzed arm is feasible by incorporating the BES into the phase-shift conditions of the movement phases that constitute the learned work support. The average work time was 59 s, with a standard deviation of 1.7 s before changing the phase-shift conditions from voice commands to BES; however, in this experiment, it was significantly reduced to 44 s with a standard deviation of 0.9 s. This result is likely because the BES from the paralyzed arm input that directly indicates the intention to move in the coordinated movements of the two hands contributed to the realization of more integrated movements with the body compared to voice information. Furthermore, compared to before the addition of this function, where the BES was solely used for operations during teaching support actions, an increased sense of unity with the body was confirmed. This is believed to be due to the enhanced execution of support actions in accordance with the intention to move using the BES for work support. In this experiment, we applied the system by replacing the default learned phase-shift condition; however, it can also be used in combination, and various forms of application can be attempted depending on the user. For example, for a patient with many involuntary signals in their paralyzed arm’s BES, a hybrid condition with other sensor information may realize phase shifting and work support according to the intention of the paralyzed arm to move. Additionally, various BES not limited to the flexion/extension of fingers, such as those from the elbow and shoulder, can be employed for the system. A system capable of these diverse applications was developed in this study. We aim to verify which form of application is more effective for different disability situations when the number of research participants increases in the future.

### 4.3 Effort to support daily tasks

In this study, we applied the system to seven tasks that were difficult for the participant in his daily life and confirmed that each task could be performed. The proposed method enables the user’s arm to perform tasks such as using tools like scissors, which are difficult for robots owing to safety and hardware limitations. Therefore, tasks that are challenging for robots alone or for individuals with a single upper-limb dysfunction alone can be accomplished through the cooperation of both the robots and the user. Additionally, the proposed method includes a framework that can learn the work support corresponding to each user and task. Hence, the system can be applied to support various tasks in the daily lives of patients, improving their quality of life by enabling them to perform tasks that were previously difficult, including those in this study, through work support that cooperates with the unaffected arm according to the user’s intention. Currently, there are some limitations to the applicable tasks, such as the inability to support cooking tasks owing to waterproofing issues. However, it is expected that more tasks will be supported by making the system waterproof and preparing attachment-type end effectors specialized for work environments such as cooking. Additionally, the gripping force is fixed according to the force learned for each task in this study. To address more diverse tasks, such as adjusting the gripping force to prevent slipping when the weight of the object increases during a task, we aim to implement a function that adjusts the gripping force in response to changes in the object’s weight. The tactile force sensor installed in our system can detect the forces applied to the gripped object and is also effective in detecting weight changes.

Through these experiments on patients with a single upper-limb dysfunction, we confirmed the feasibility and usefulness of various cooperative tasks and work support that reflect the user’s intention using the developed cybernic robot hand-arm.

## 5 Conclusion

In order to provide support for upper-limb tasks in daily life for patients with a single upper-limb dysfunction, a challenge that has been difficult to overcome, we proposed and developed a cybernic robot hand-arm with the following features: 1) input of BES that reflects the intention to move from the paralyzed arm, motion information from the unaffected arm, and voice-based intention information; 2) autonomous control of support movements according to the unaffected arm and work phase; 3) a control system that integrates voluntary and autonomous control by combining 1) and 2), allowing for smooth work support in cooperation with the unaffected arm reflecting intention as a part of the body; and 4) a learning function to provide such work support across various tasks in daily use. The system learns to support new tasks by working with the user through an operating function that does not require operation by the unaffected arm. The system divides the support actions into movement phases and learns the phase-shift conditions from the sensor information on the user’s intention. After learning, the system autonomously performs learned support actions through voluntary phase shifts based on input of the user’s intention via BES from the paralyzed arm, the unaffected arm’s motion, and voice, enabling smooth collaborative work with the unaffected arm. We conducted a pilot study involving three patients and confirmed that the system could learn and provide smooth work support in cooperation with the unaffected arm that reflected the user’s intention, successfully completing tasks that had been difficult for them. Additionally, the questionnaire confirmed that, subjectively, cooperative work according to the user’s intention was achieved, and that the work time was within a range feasible for daily life. These results confirmed the feasibility and usefulness of the proposed method. Additionally, it was observed from the questionnaire that participants who used BES for the control system as their intention information perceived the system as part of their body. In future research, we aim to increase the number of patients and tasks to which the proposed method is applied in order to further verify its range of applicability and usefulness, as well as the phenomenon of sensory integration with the body.

## Data Availability

The original contributions presented in the study are included in the article/Supplementary Material; further inquiries can be directed to the corresponding author.
