# Supplementary material for: Cybernic robot hand-arm that realizes cooperative work as a new hand-arm for people with a single upper-limb dysfunction
Source: Front Robot AI. 2024 Oct 22;11:1455582. doi: 10.3389/frobt.2024.1455582 (PMC11535860; doi:10.3389/frobt.2024.1455582)
Supplement: Supplementary file 3 [file DataSheet1.pdf]

Supplementary Material

1 Supplementary Data

The learned data for work support obtained from each experiment are listed below.

1.1 Experiment on learning and implementation of work support cooperated with the unaffected arm.

Supplementary Table 1. Learning result of the "opening a medicine package" task with the participant A.

Phase	Movement	Phase-shift condition
0	Move A:<br>Position (350, 0, 100) [mm]<br>Posture (0, 90, 0) [deg]	Automatic
1	Grip A: 3.4 [N]	ΔDistance1 ≦ -102 [mm]
2	Release A	Norm1 ≧ 2.6 [mm]<br>Norm2 ≧ 2.1 [mm]<br>-30 ≦ Angle1 ≦ 30 [deg]<br>-30 ≦ Angle2 ≦ 30 [deg]
3	Move B:<br>Position (250, 0, 400) [mm]<br>Posture (0, 90, 0) [deg]	Automatic

Position (x, y, z), Posture (Roll, Pitch, Yaw)

Supplementary Table 2. Learning result of the "opening a PET bottle" task with the participant A.

Phase	Movement	Phase-shift condition
0	Move A:<br>Position (350, 0, 100) [mm]<br>Posture (0, 90, 0) [deg]	Automatic
1	Grip A: 4.7 [N]	ΔDistance1 ≦ -110 [mm]<br>ΔDistance2 ≦ -128 [mm]
2	Release A	Norm1 ≧ 3.2 [mm]<br>Norm2 ≧ 1.8 [mm]<br>-48 ≦ Angle1 ≦ 12 [deg]<br>-58 ≦ Angle2 ≦ 2 [deg]
3	Move B:<br>Position (250, 0, 400) [mm]<br>Posture (0, 90, 0) [deg]	Automatic

Position (x, y, z), Posture (Roll, Pitch, Yaw)

Supplementary Table 3. Learning result of the "eating a jelly cup" task with the participant A. (Same as Manuscript Table.1)

Phase	Movement	Phase-shift condition
0	Move A:<br>Position (350, 0, 30) [mm]<br>Posture (0, 90, 0) [deg]	Automatic
1	Grip A: 4.4 [N]	ΔDistance1 ≦ -112 [mm]<br>ΔDistance2 ≦ -119 [mm]
2	Move B:<br>Position (350, -100, 180) [mm]<br>Posture (0, 90, 0) [deg]	Voice command
3	Move C:<br>Position (350, -100, 180) [mm]<br>Posture (0, 90, 30) [deg]	Voice command
4	Move D:<br>Position (350, 0, 30) [mm]<br>Posture (0, 90, 0) [deg]	Voice command
5	Release A	Norm1 ≧ 3.1 [mm]<br>Norm2 ≧ 2.0 [mm]<br>-57 ≦ Angle1 ≦ 3 [deg]<br>-62 ≦ Angle2 ≦ -2 [deg]
6	Move E:<br>Position (250, 0, 400) [mm]<br>Posture (0, 90, 00) [deg]	Automatic

Position (x, y, z), Posture (Roll, Pitch, Yaw)

Supplementary Table 4. Learning result of the "opening a medicine package" task with the participant B.

Phase	Movement	Phase-shift condition
0	Move A:<br>Position (400, 0, 100) [mm]<br>Posture (0, 90, 0) [deg]	Automatic
1	Grip A: 4.9 [N]	ΔDistance1 ≦ -99 [mm]
2	Release A	Norm1 ≧ 1.9 [mm]<br>-30 ≦ Angle1 ≦ 30 [deg]
3	Move B:<br>Position (250, 0, 400) [mm]<br>Posture (0, 90, 0) [deg]	Automatic

Position (x, y, z), Posture (Roll, Pitch, Yaw)

Supplementary Table 5. Learning result of the "opening a PET bottle" task with the participant B.

Phase	Movement	Phase-shift condition
0	Move A:<br>Position (400, 0, 100) [mm]<br>Posture (0, 90, 0) [deg]	Automatic
1	Grip A: 5.3 [N]	ΔDistance1 ≦ -114 [mm]<br>ΔDistance2 ≦ -125 [mm]
2	Release A	Norm1 ≧ 6.0 [mm]<br>Norm2 ≧ 4.5 [mm]<br>-48 ≦ Angle1 ≦ 12 [deg]<br>-47 ≦ Angle2 ≦ 13 [deg]
3	Move B:<br>Position (250, 0, 400) [mm]<br>Posture (0, 90, 0) [deg]	Automatic

Position (x, y, z), Posture (Roll, Pitch, Yaw)

Supplementary Table 6. Learning result of the "eating a jelly cup" task with the participant B.

Phase	Movement	Phase-shift condition
0	Move A:<br>Position (360, 0, 30) [mm]<br>Posture (0, 90, 0) [deg]	Automatic
1	Grip A: 2.1 [N]	ΔDistance1 ≦ -117 [mm]<br>ΔDistance2 ≦ -126 [mm]
2	Move B:<br>Position (310, 0, 140) [mm]<br>Posture (0, 90, 0) [deg]	Voice command
3	Move C:<br>Position (310, 0, 190) [mm]<br>Posture (0, 90, -30) [deg]	Voice command
4	Move D:<br>Position (350, 0, 30) [mm]<br>Posture (0, 90, 0) [deg]	Voice command
5	Release A	Norm1 ≧ 3.9 [mm]<br>Norm2 ≧ 5.3 [mm]<br>-30 ≦ Angle1 ≦ 30 [deg]<br>-13 ≦ Angle2 ≦ 47 [deg]
6	Move E:<br>Position (250, 0, 400) [mm]<br>Posture (0, 90, 00) [deg]	Automatic

Position (x, y, z), Posture (Roll, Pitch, Yaw)

Supplementary Table 7. Learning result of the "opening a medicine package" task with the participant C.

Phase	Movement	Phase-shift condition
0	Move A:<br>Position (350, 0, 100) [mm]<br>Posture (0, 90, 0) [deg]	Automatic
1	Grip A: 5.9 [N]	ΔDistance1 ≦ -91 [mm]
2	Release A	Norm1 ≧ 1.7 [mm]<br>-30 ≦ Angle1 ≦ 30 [deg]
3	Move B:<br>Position (250, 0, 400) [mm]<br>Posture (0, 90, 0) [deg]	Automatic

Position (x, y, z), Posture (Roll, Pitch, Yaw)

Supplementary Table 8. Learning result of the "opening a PET bottle" task with the participant C.

Phase	Movement	Phase-shift condition
0	Move A:<br>Position (350, 0, 100) [mm]<br>Posture (0, 90, 0) [deg]	Automatic
1	Grip A: 5.9 [N]	ΔDistance1 ≦ -119 [mm]<br>ΔDistance2 ≦ -119 [mm]
2	Release A	Norm1 ≧ 3.3 [mm]<br>Norm2 ≧ 1.2 [mm]<br>-30 ≦ Angle1 ≦ 30 [deg]<br>-30 ≦ Angle2 ≦ 30 [deg]
3	Move B:<br>Position (250, 0, 400) [mm]<br>Posture (0, 90, 0) [deg]	Automatic

Position (x, y, z), Posture (Roll, Pitch, Yaw)

Supplementary Table 9. Learning result of the "eating a jelly cup" task with the participant C.

Phase	Movement	Phase-shift condition
0	Move A:<br>Position (350, 0, 30) [mm]<br>Posture (0, 90, 0) [deg]	Automatic
1	Grip A: 4.6 [N]	ΔDistance1 ≦ -101 [mm]<br>ΔDistance2 ≦ -119 [mm]
2	Move B:<br>Position (350, 50, 230) [mm]<br>Posture (0, 90, 0) [deg]	Voice command
3	Move C:<br>Position (350, 50, 230) [mm]<br>Posture (0, 90, -30) [deg]	Voice command
4	Move D:<br>Position (350, 0, 30) [mm]<br>Posture (0, 90, 0) [deg]	Voice command
5	Release A	Norm1 ≧ 4.1 [mm]<br>Norm2 ≧ 1.5 [mm]<br>-30 ≦ Angle1 ≦ 30 [deg]<br>-30 ≦ Angle2 ≦ 30 [deg]
6	Move E:<br>Position (250, 0, 400) [mm]<br>Posture (0, 90, 00) [deg]	Automatic

Position (x, y, z), Posture (Roll, Pitch, Yaw)

1.2 Effort to support daily tasks

Supplementary Table 10. Learning result of the "opening a packaged bread" task.

Phase	Movement	Phase-shift condition
0	Move A:<br>Position (350, 0, 100) [mm]<br>Posture (0, 90, 0) [deg]	Automatic
1	Grip A: 0.9 [N]	ΔDistance1 ≦ -120 [mm]<br>ΔDistance2 ≦ -124 [mm]
2	Release A	Norm1 ≧ 3.1 [mm]<br>Norm2 ≧ 2.8 [mm]<br>-47 ≦ Angle1 ≦ 13 [deg]<br>-58 ≦ Angle2 ≦ 2 [deg]
3	Move B:<br>Position (250, 0, 400) [mm]<br>Posture (0, 90, 0) [deg]	Automatic

Position (x, y, z), Posture (Roll, Pitch, Yaw)

Supplementary Table 11. Learning result of the "putting toothpaste on a toothbrush" task.

Phase	Movement	Phase-shift condition
0	Move A:<br>Position (320, 0, 100) [mm]<br>Posture (0, 90, 0) [deg]	Automatic
1	Grip A: 5.1 [N]	ΔDistance1 ≦ -112 [mm]
2	Release A	Norm1 ≧ 1.8 [mm]<br>Norm2 ≧ 1.3 [mm]<br>-30 ≦ Angle1 ≦ 30 [deg]<br>-30 ≦ Angle2 ≦ 30 [deg]
3	Move B:<br>Position (250, 0, 400) [mm]<br>Posture (0, 90, 0) [deg]	Automatic

Position (x, y, z), Posture (Roll, Pitch, Yaw)

Supplementary Table 12. Learning result of the "stapling papers together" task.

Phase	Movement	Phase-shift condition
0	Move A:<br>Position (320, 0, 150) [mm]<br>Posture (0, 90, 0) [deg]	Automatic
1	Grip A: 3.9 [N]	ΔDistance1 ≦ -122 [mm]<br>ΔDistance2 ≦ -85 [mm]
2	Release A	Norm1 ≧ 2.8 [mm]<br>Norm2 ≧ 1.2 [mm]<br>-30 ≦ Angle1 ≦ 30 [deg]<br>-30 ≦ Angle2 ≦ 30 [deg]
3	Move B:<br>Position (250, 0, 400) [mm]<br>Posture (0, 90, 0) [deg]	Automatic

Position (x, y, z), Posture (Roll, Pitch, Yaw)

Supplementary Table 13. Learning result of the "cutting paper with scissors" task.

Phase	Movement	Phase-shift condition
0	Move A:<br>Position (300, 0, 200) [mm]<br>Posture (0, 90, 0) [deg]	Automatic
1	Grip A: 3.3 [N]	ΔDistance1 ≦ -113 [mm]<br>ΔDistance2 ≦ -108 [mm]
2	Move B:<br>Position (300, 0, 200) [mm]<br>Posture (0, 90, -30) [deg]	Voice command
3	Release A	Norm1 ≧ 2.3 [mm]<br>Norm2 ≧ 2.2 [mm]<br>-30 ≦ Angle1 ≦ 30 [deg]<br>-30 ≦ Angle2 ≦ 30 [deg]
4	Grip B: 3.3 [N]	ΔDistance1 ≦ -125 [mm]<br>ΔDistance2 ≦ -118 [mm]
5	Move C:<br>Position (300, 0, 200) [mm]<br>Posture (0, 90, 0) [deg]	Voice command
6	Release B	Norm1 ≧ 2.2 [mm]<br>Norm2 ≧ 2.3 [mm]<br>-72 ≦ Angle1 ≦ -12 [deg]<br>-77 ≦ Angle2 ≦ -17 [deg]
7	Move D:<br>Position (250, 0, 400) [mm]<br>Posture (0, 90, 00) [deg]	Automatic

Position (x, y, z), Posture (Roll, Pitch, Yaw)

Supplementary Table 14. Learning result of the "administering eye drops" task.

Phase	Movement	Phase-shift condition
0	Move A:<br>Position (350, 0, 100) [mm]<br>Posture (0, 90, 0) [deg]	Automatic
1	Grip A: 3.6 [N]	ΔDistance1 ≦ -44 [mm]
2	Release A	Norm1 ≧ 4.3 [mm]<br>Norm2 ≧ 3.2 [mm]<br>-41 ≦ Angle1 ≦ 19 [deg]<br>-30 ≦ Angle2 ≦ 30 [deg]
3	Grip B: 3.6 [N]	ΔDistance1 ≦ -80 [mm]
4	Release B	Norm1 ≧ 3.0 [mm]<br>Norm2 ≧ 2.1 [mm]<br>-30 ≦ Angle1 ≦ 30 [deg]<br>-30 ≦ Angle2 ≦ 30 [deg]
5	Move B:<br>Position (250, 0, 400) [mm]<br>Posture (0, 90, 00) [deg]	Automatic

Position (x, y, z), Posture (Roll, Pitch, Yaw)

Supplementary Table 15. Learning result of the "applying lip balm" task.

Phase	Movement	Phase-shift condition
0	Move A:<br>Position (320, 0, 100) [mm]<br>Posture (0, 90, 0) [deg]	Automatic
1	Grip A: 5.4 [N]	ΔDistance1 ≦ -81 [mm]
2	Release A	Norm1 ≧ 2.4 [mm]<br>Norm2 ≧ 2.2 [mm]<br>-57 ≦ Angle1 ≦ 3 [deg]<br>-30 ≦ Angle2 ≦ 30 [deg]
3	Grip B: 5.4 [N]	ΔDistance1 ≦ -75 [mm]
4	Release B	Norm1 ≧ 1.6 [mm]<br>Norm2 ≧ 2.0 [mm]<br>-30 ≦ Angle1 ≦ 30 [deg]<br>-30 ≦ Angle2 ≦ 30 [deg]
5	Move B:<br>Position (250, 0, 400) [mm]<br>Posture (0, 90, 00) [deg]	Automatic

Position (x, y, z), Posture (Roll, Pitch, Yaw)

Supplementary Table 16. Learning result of the "applying medicine" task.

Phase	Movement	Phase-shift condition
0	Move A:<br>Position (350, 0, 100) [mm]<br>Posture (0, 90, 0) [deg]	Automatic
1	Grip A: 2.4 [N]	ΔDistance1 ≦ -90 [mm]<br>ΔDistance2 ≦ -87 [mm]
2	Move B:<br>Position (350, 0, 100) [mm]<br>Posture (0, 90, 90) [deg]	Voice command
3	Move C:<br>Position (350, 0, 100) [mm]<br>Posture (0, 90, 0) [deg]	Voice command
4	Release A	Norm1 ≧ 5.5 [mm]<br>Norm2 ≧ 4.1 [mm]<br>-50 ≦ Angle1 ≦ 10 [deg]<br>-30 ≦ Angle2 ≦ 30 [deg]
5	Move D:<br>Position (250, 0, 400) [mm]<br>Posture (0, 90, 00) [deg]	Automatic

Position (x, y, z), Posture (Roll, Pitch, Yaw)
